# Enhancing Interprofessional Student Training in Age-Friendly Care: Outcomes Mobile Health and Wellness Program

**DOI:** 10.1093/geroni/igaf122.274

**Published:** 2025-12-31

**Authors:** Rachel Regal, Kimberly Battle, Katherine Falls, Genevieve Beaird, Emily Peron, Ashley Staton, Jacquelin Daniel, Glenda Watkins

**Affiliations:** Virginia Commonwealth University, Richmond, Virginia, United States; Virginia Commonwealth University, Richmond, Virginia, United States; Virginia Commonwealth University, Richmond, Virginia, United States; Virginia Commonwealth University, Richmond, Virginia, United States; Virginia Commonwealth University School of Pharmacy, Richmond, Virginia, United States; Virginia Commonwealth University, Richmond, Virginia, United States; Virginia Commonwealth University School of Nursing, Richmond, Virginia, United States; Virginia Commonwealth University College of Health Professions, Richmond, Virginia, United States

## Abstract

The Mobile Health and Wellness Program (MHWP) brings health promotion and care coordination services to medically underserved communities while training interprofessional students in age-friendly care. Since MHWP was founded in 2012, over 2700 health professions students from nursing, medicine, pharmacy, and other allied health programs have trained onsite. Students engage with interprofessional team visits and faculty debriefings, online didactic modules based in 4Ms care, morning topic discussions, and orientation simulation. Student outcomes from two mixed methods studies will be presented that demonstrate pre/post semester gains across attitudes, behaviors, and competencies. First, for students placed in older adult affordable housing building sites and a community wellness center (n = 166), students reported reduced personalized aging anxiety, increased collective affinity for older adults, and fewer negative ageist behaviors (paired t-tests, p<.05). Students also reported more favorable attitudes towards work with the medically underserved and enhanced interprofessional readiness. Second, for nursing students placed at medically underserved sites including mobile vans and rural locations (n = 174), students reported confidence gains across eight competency domains of public health nursing (Wilcox Signed-Rank Test, p<.001). Qualitative themes from student focus groups highlight the strengths of the MHWP training model in facilitating positive student outcomes, including a low-stakes learning setting to build confidence, time for and value on relationship and rapport building, inclusion of social drivers for health as part of shared health promotion, and interprofessional practice based in cultural humility. Implications for student training programs in age-friendly care with medically underserved communities will be shared.

